# Effects of immunomodulation in classic infantile Pompe patients with high antibody titers

**DOI:** 10.1186/s13023-019-1039-z

**Published:** 2019-03-22

**Authors:** E. Poelman, M. Hoogeveen-Westerveld, J. M. P. van den Hout, R. G. M. Bredius, A. C. Lankester, G. J. A. Driessen, S. S. M. Kamphuis, W. W. M. Pijnappel, A. T. van der Ploeg

**Affiliations:** 1 Center for Lysosomal and Metabolic Diseases, Erasmus MC University Medical Center, P.O. BOX 2060, 3000 CB Rotterdam, The Netherlands; 2 Department of Pediatrics, Leiden Medical University Center, Leiden, The Netherlands; 3 Department of Pediatrics, Juliana Children’s Hospital, The Hague, The Netherlands; 4 Department of Pediatric Rheumatology, Erasmus MC University Medical Center, Rotterdam, The Netherlands

**Keywords:** Pompe disease, ERT, Antibodies, Immunomodulation, Bortezomib, Cross-reactive immunologic material (CRIM)

## Abstract

**Purpose:**

To evaluate whether immunomodulation can eliminate high sustained antibody levels, and thereby improve clinical outcome in classic infantile Pompe patients receiving enzyme replacement therapy (ERT) with recombinant human alpha-glucosidase (rhGAA).

**Methods:**

Three patients (two cross-reactive immunologic material (CRIM) negative) with high sustained antibodies received a three-week treatment protocol with Rituximab and Bortezomib, followed by daily Rapamycin and monthly IVIG. Patients received 40 mg/kg/week rhGAA. Antibody titers were measured using ELISA. Neutralizing effects on cellular uptake were determined. Clinical efficacy was measured in terms of (ventilator-free) survival, reduction in left ventricular mass index (LVMI) and improvement in motor function.

**Results:**

Before immunomodulation anti-rhGAA antibody titers ranged from 1:156,250 to 1:781,250 and at last assessment from 1:31,250 to 1:156,250. Neutralizing effects of anti-rhGAA antibody titers (observed in two patients) disappeared. Infusion-associated reactions were no longer present. Immunomodulation resulted in substantial increases of aspartate transaminase, alanine transaminase, and creatine kinase levels. The two CRIM-negative patients who could walk at start of immunomodulation maintained their ability to walk; the patient who had lost this ability did not regain it.

**Conclusions:**

To some extent, the immunomodulation protocol used in our study reduced antibody titers, but it did not eliminate them. Overall, there have been few reports on secondary immunomodulation, and various protocols have been applied. Future research should seek to identify the most successful immunomodulation protocol in patients with high sustained titers.

**Electronic supplementary material:**

The online version of this article (10.1186/s13023-019-1039-z) contains supplementary material, which is available to authorized users.

## Background

Pompe disease (Glycogen Storage Disease type II, OMIM #232300), an autosomal recessive lysosomal storage disorder caused by deficiency of acid-α-glucosidase (GAA), results in lysosomal glycogen accumulation in all cell types, but mainly in muscle cells [1]. Pompe disease presents as a spectrum of clinical phenotypes, the classic infantile form being the most severe form [2]. Classic infantile patients have less than 1% of enzyme activity in fibroblasts and present with hypertrophic cardiomyopathy (HCM), progressive generalized muscle weakness, and respiratory difficulties. Without treatment, patients die within the first year of life due to cardiorespiratory failure [3–5].

Enzyme replacement therapy (ERT) with recombinant human alpha-glucosidase (rhGAA, alglucosidase alfa, Myozyme) has improved prognosis for patients by improving survival and improving motor outcome. [6, 7]. Clinical response varies greatly between patients [6, 8–11]. A higher dose of ERT has been shown to positively influence patients’ outcome [6, 12, 13]. Antibodies to rhGAA may counteract positive effects of ERT by neutralizing its activity or preventing cellular uptake [12, 14–16]. Cross-reactive immunologic material (CRIM negative) patients (who produce no GAA protein) are more likely to form higher anti-rhGAA antibodies titers than CRIM-positive infantile patients (who produce inactive GAA protein). Generally, CRIM-negative patients have been reported to respond poorly to ERT [14, 16–18]. Despite reports that antibody formation can be prevented successfully by primary immunomodulation (i.e., using a combination of Rituximab (RTX), Methotrexate (MTX) and intravenous immunoglobulin (IVIG) before the first ERT dose) [8, 19–21], some patients still develop high anti-rhGAA antibody titers [21–23].

There have also been attempts at secondary immunomodulation, i.e., eliminating anti-rhGAA antibodies in patients who have developed high titers during ERT. The best reported results involved a protocol using Bortezomib, a proteasome inhibitor that induces apoptosis in plasma cells by dysregulating signaling cascades [24]. So far, the data that has been published is on very few patients, whose outcome also vary [19, 20, 25–28]. We studied the effects of secondary immunomodulation on anti-rhGAA antibody titer formation, the cellular uptake of GAA, the depletion and repopulation of B cells, and clinical outcome using a protocol combining Rituximab, Bortezomib, Rapamycin and IVIG.

## Methods

### Patients and treatment protocol

We included three patients with classic infantile Pompe disease who had high sustained anti-rhGAA antibodies (≥1:31.500) and whose quality of movement and/or motor performance raised concerns. Two patients did not receive immunomodulation previously, while (patient 2 did [23]). Classic infantile Pompe disease was defined as symptoms of muscle weakness within 6 months after birth, the presence of HCM, complete deficiency of α-glucosidase in fibroblasts (< 1% of normal values), and two very severe mutations in the GAA gene. Patients were already participating in an ongoing study into the effects of ERT, study protocols had been approved by the Institutional Review Board. Written informed consent was obtained from the parents. ERT was dosed at 40 mg/kg/week.

### Immunomodulation protocol

Our immunomodulation protocol for patients with high titers was derived from protocols published by Messinger et al., Banugaria et al. and Elder et al. [19, 22, 24]. The following immunomodulation regimen was applied: 3 weekly infusions of RTX 375 mg/m^2^; 6 twice-weekly doses of Bortezomib 1.3 mg/m^2^; monthly IVIG (first dose 1.0 g/kg; subsequent doses of 0.5 g/kg); Rapamycin was commenced at week 4 (10–20 kg 1.0–1.5 mg/day; 20–30 kg 1.5–2.0 mg/day; double dose on first day of Rapamycin treatment). Dose was adjusted on the basis of serum Rapamycin levels (normal range 4–12 μg/l). To reduce the risk of infections, all patients received Azithromycin prophylactically. Regular blood analysis consisted of determining the number of B cells and the levels of aspartate transaminase (AST), alanine transaminase (ALT), creatine kinase (CK), and gamma globulin (IgG, IgM, IgA).

### Antibody titer and neutralizing effects

Blood samples were drawn at regular intervals and stored at − 80 °C until analysis. Anti-rhGAA antibody titers were determined by enzyme-linked immunosorbent assay (ELISA) as described earlier [16, 17]. Experiments were performed in duplicate and assays were repeated at least twice. Our figures present the highest titers measured. The neutralizing effects of antibodies were determined at least twice per patient by studying their effect on cellular uptake in vitro. Fibroblasts from a patient homozygous for the 525delT mutation fully deficient in acid α-glucosidase production were seeded in 24-well tissue-culture plates and maintained at 37 °C in Ham’s F10 medium supplemented with 10% FCS and antibiotics. To measure the uptake of alglucosidase alfa, we added Pipes to the medium in a final concentration of 3 mM to make the medium slightly acidic (pH 6.8). The enzyme was added in an amount equivalent to 200 nmol MUGlc/h per 200 μL medium. Finally, 20 μL of the patients’ sera were added. Uptake of alglucosidase alfa was measured in cell homogenates. MUGlc was used as substrate”. As control the same experiment was performed without addition of 20 μL of the patients’ sera. GAA activity was expressed as percentage of control. The experiment was performed in duplicate [16, 17].

### Clinical outcome measures

Standardized assessments were performed at start of ERT and every three months thereafter [6], and also at start of immunomodulation. Clinical outcome parameters were (ventilator-free) survival, left ventricular mass index (LVMI, where an LVMI Z-score ≥ +2SD was defined as abnormal); pulmonary function tests; and motor function assessed by Alberta Infant Motor Scale (AIMS); Bayley Scales of Infant Development II (BSID-II); 10-m run test, and 6-min walk test (6MWT). Infusion-associated reactions (IARs) were recorded [29–34].

## Case reports

### Patient 1

Patient 1 (CRIM-positive) started ERT at 2.4 months (Table 1). At birth she had presented with persistent tachypnea and HCM attributed initially to gestational diabetes in her mother. At the time of diagnosis she had severe hypotonia and a prominent head lag. When prone she could not lift her head from the surface, and anti-gravity movements of the limbs were not observed (AIMS score was 1). She was still able to drink (weight 6.5 kg + 2.0 SD for Dutch children) and did not require nasogastric tube (NGT) feeding. During ERT, LVMI normalized, with the LVMI z-score decreasing from 22.5 to 1.75 during the first 9 months of ERT (Fig. 3e). She learned to walk unsupported at the age of 15 months. At the age of 2.5 years she developed a transient right-sided facial nerve palsy elicited by a herpes simplex viral infection. From then on she experienced frequent airway and urinary tract infections, accompanied by transient periods of poorer motor functioning. She maintained the ability to walk until the age of 6 years. From the age of 6.1 years, motor function started to decline. At the age of 6.4 years she was no longer able to stand unsupported.

**Table 1:** Patient Characteristics

	Patient 1	Patient 2^a^	Patient 3
Baseline and initial response			
Age at start (in months)	2.4	5.8	1.9
Mutations	c.2481 + 102_2646 + 31del538	c.del525T	c.del525T
	c.2481 + 102_2646 + 31del538	c.del525T	c.del525T
CRIM status	Positive	Negative	Negative
Ventilatory support	No	No	No
LVMI at start in g/m2 (z-score)	237 (22.5)	265 (26.1)	200 (17.8)
Time to LVMI normalization (z-score)	9 months (1.75)	6 months (1.43)	9 months (0.1)
Age pull to stand (in months)	11.6	14.8	9.2
Age walking (in months)	15	21.3	11.7
NGT at start	No	Yes	Yes
Age at which NGT ended (in months)	N.A	21	9
Total number of IARs (total severe IARs)	70 (6)	22 (5)	16 (0)
Age at last IAR (in years)	4.0	3.5	2.1
At start of secondary immunomodulation			
Age in years	6.6	3.5	2.3
Ventilatory support	No	No	No
LVMI in g/m2 (z-score)	70.6 (0.4)	63.2 (0.5)	83.9 (3.1)
Best motor function	Sitting	Walking	Walking
Antibody titer	1:156,250	1:156,250	1:781,250
Enzyme activity in cell lysates	50%	60%	100%
At study end			
Age in years	9.1	5.6	3.8
Ventilatory support	No	No	No
LVMI in g/m2 (z-score)	82.5 (1.3)	65 (0.7)	55 (−0.5)
Best motor function	Sitting	Walking	Walking
NGT/PEG (age in years)	Yes (7.0)	No	No
Last antibody titer (time since last RTX in years)	1:31,250 (0.5)	1:31,250 (2)	1:1561,250 (1.5)
Enzyme activity in cell lysates	100%	100%	100%
B-cell normalization/time since last RTX in months	Yes/14 No/5^c^	Yes/6	Yes/3
Last B-cell level^b^	0	0.85*10^9^/L	0.48*10^9^/L
IARs since start of immunomodulation	No	No	No

*CRIM* cross-reactive immunologic material, *LVMI* left ventricular mass index, *NGT* nasogastric tube, *IAR* infusion-associated reaction, *PEG* percutaneous endoscopic gastrostomy tube, *RTX* Rituximab

^a^Patient 2 initially received immunomodulation in an ERT naïve setting.

^b^B- cell normal range; for age 2–5 years normal range of 0.2–2.1*10E9, for age 5–10 years normal range of 0.2–1.6*10E9

^c^After an initial round of immunomodulation patient 2 received a second round of immunomodulation 2 years later because of high rhGAA antibodies

### Patient 2

Patient 2 (CRIM-negative) started ERT at 5.8 months (Table 1). At 5 months he was hospitalized due to feeding difficulties and muscle weakness accompanied by HCM. At time of diagnosis he was able to lift his limbs from the surface, but could not roll over. Due to insufficient oral intake, NGT feeding was started. Primary immunomodulation was started before the first ERT dose [23]. During ERT, LVMI normalized, with the LVMI z-score decreasing from 26.1 to 1.4 during the first 6 months of ERT (Fig. 3e). NGT feeding could be stopped at age of 21 months. He learned to walk unsupported at 21 months. After a fall he lost the ability to walk for 4 months (age 2.5 years), but then regained it without intervention. Due to the concerns raised by his quality of movement and the reoccurrence of IARs, a second round of immunomodulation was initiated.

### Patient 3

Patient 3 (CRIM-negative) started ERT at 1.9 months (Table 1). There were feeding difficulties from birth onwards. Shortly after a chest X-ray at the age of 5 weeks had revealed HCM, she was diagnosed with Pompe disease. At time of diagnosis she could lift her limbs from the surface, could turn her head when in prone position, and could take some support on her legs. Due to insufficient intake, an NGT was placed. After start of ERT, LVMI normalized, with LVMI z-score decreasing from 17.8 (LVMI 200 g/m^2^) to 0.1 during the first 9 months of ERT (Fig. 3). NGT feeding could be stopped at the age of 9 months. She learned to walk unsupported at the age of 11 months, and obtained the maximum AIMS score of 58 at the age of 12 months (Fig. 3). After her first birthday, she gradually started to perform more poorly than age-related peers (BSID II scores, Fig. 3e). She also developed a Gower’s sign and her calves became hypertrophic. LVMI increased slightly (LVMI 80.7 g/m^2^, z-score 2.7) without functional consequences.

## Results

### Effects of immunomodulation on B cells

After RTX treatment B cells became depleted in all patients (Table 1). In patients 1, 2 and 3 time to B-cell recovery was 1.2 years, 6 and 3 months, respectively. During B-cell depletion all patients received IVIG. Patient 1 who received the first round at the age of 6.6 years received a second round of immunomodulation at 8.5 years. At study end there was no B-cell recovery.

### Anti-rhGAA antibody titers before and after immunomodulation

**Fig. 1:**
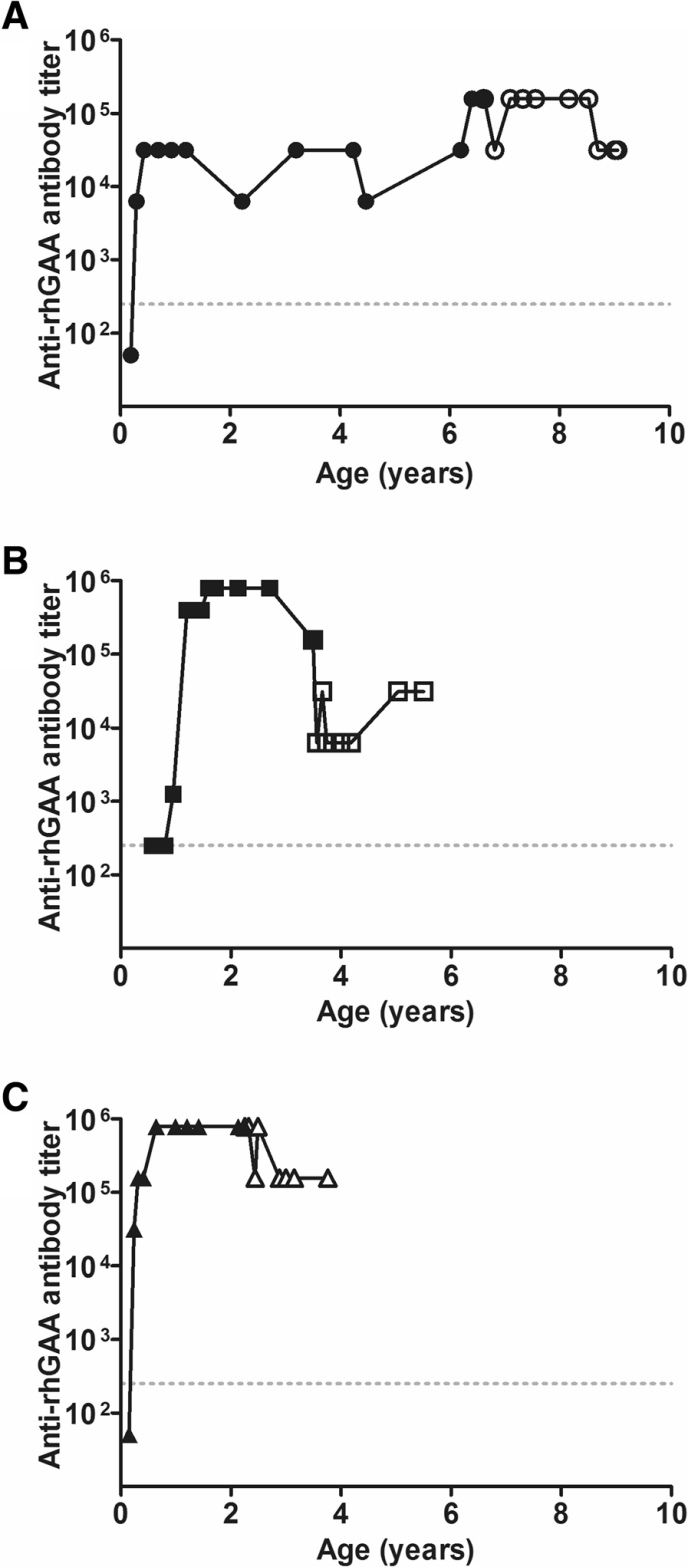
Anti-rhGAA antibody titers. Anti-rhGAA antibody titers per patient during follow-up. Panel **a** is patient 1. Panel **b** is patient 2. Panel **c** is patient 3. Anti-rhGAA antibody titers before immunomodulation are shown as closed symbols. The open symbols represent titers after immunomodulation

Anti-rhGAA antibody titers are shown in Figs. 1 (from start of ERT) and 2 (after immunomodulation). In patient 1, anti-rhGAA antibodies were first detected at one month of ERT (titer 1:250, Fig. 1). This increased to a maximum titer of 1:31,250, which was maintained between the ages of 0.4 and 6.2 years. At start of secondary immunomodulation at 6.4 years, her titer was 1:156,250. In patient 2, who had started primary immunomodulation before start of ERT, anti-rhGAA antibodies were first detected at 5.5 months of ERT (titer 1:1250). These increased to the highest maximum titer of 1:800,000 between the ages of 1.6 and 2.7 years [23]. This patient had received MTX in a dose of 0.5 mg/kg/week until 5 days before start of secondary immunomodulation. At start of secondary immunomodulation at 3.5 years, his titer was 1:156,250. In patient 3, anti-rhGAA antibodies were first detected at one month of ERT (1: 31,250). These increased to a titer of 1:781,250, which was maintained until start of secondary immunomodulation at the age of 2.3 years.

**Fig. 2:**
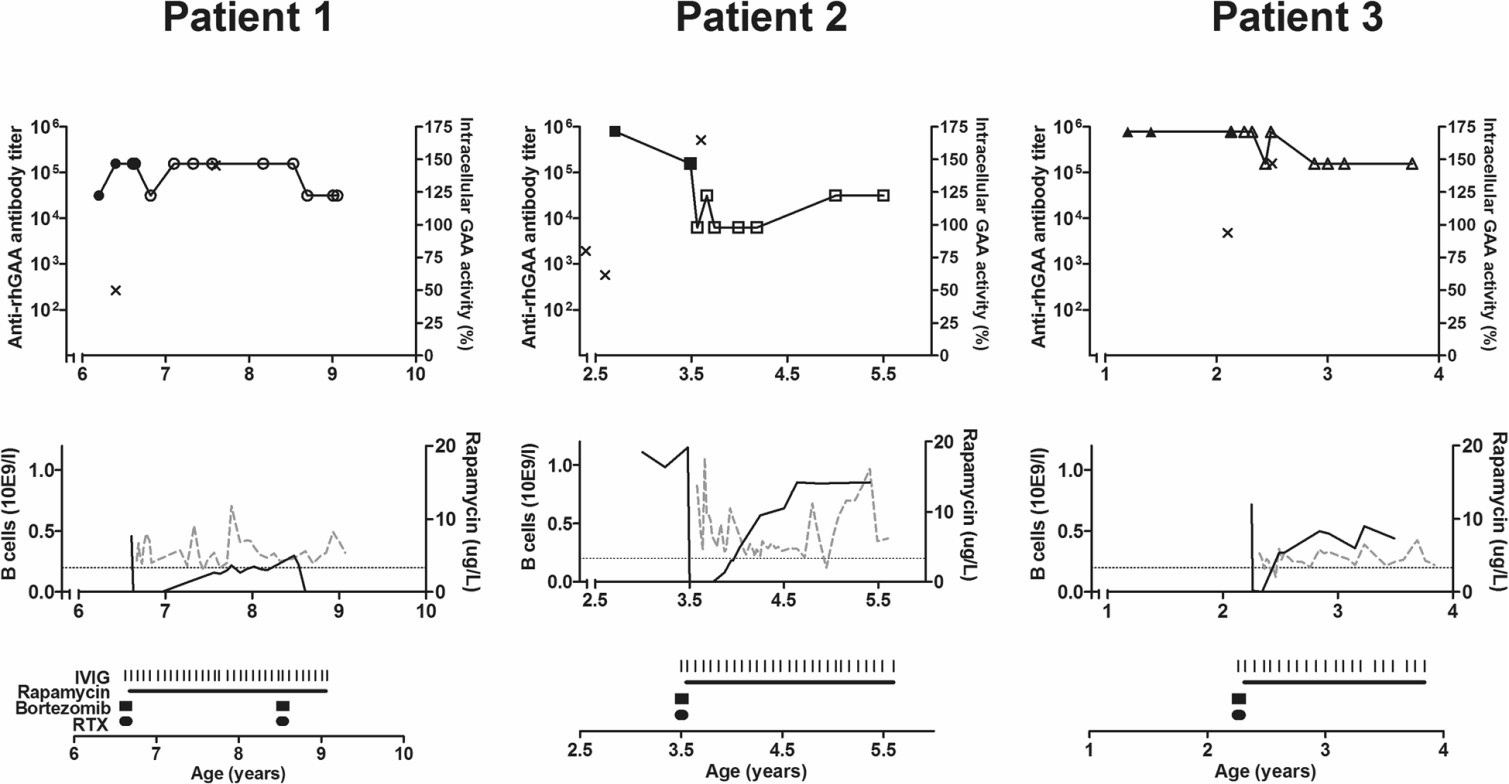
Effects of immunomodulation. Each column represents a single patient. *Upper row:* Anti-rhGAA antibody titer in detail after immunomodulation, as shown previously in Fig. 1 (line with symbols on the left axis), and neutralizing effects of anti-rhGAA antibodies (crosses, on the right axis). *Middle row:* Serum B-cell levels per patient (black line on the left axis) and Rapamycin serum levels per patient (grey dashed line on the right axis). Dotted grey line represents the lower level of normal for B cells for age, which is 0.2*10E9/l. *Bottom row:* Immunomodulation treatment per patient. Verticals stripes represent each individual IVIG infusion. Horizontal lines represent the period in which Rapamycin taken. Squares represent each cycle of 6 Bortezomib infusions. Circles represent each cycle of 3 Rituximab infusions

The immunomodulation schedule per patient is shown in Fig. 2, as well as the effects on anti-rhGAA antibody titer. In patient 1 anti-rhGAA antibody titers decreased from 1:156,250 to 1:31,250 one month after start of immunomodulation, but rose again to 1:156,250 a month later. The high sustained titers were the reason for starting a second round of immunomodulation 2 years later. Titers decreased to 1:31,250. In patient 2, anti-rhGAA antibody titers decreased from 1:156,250 to 1:6250; his last titer was 1:31,250. In patient 3, anti-rhGAA antibody titers decreased from 1:781,250 to 1:156,250.

### Neutralizing effects of antibodies

To test for neutralizing effects of anti-rhGAA antibodies, GAA-deficient fibroblasts were incubated with alglucosidase alpha plus patients’ serum. Enzyme activity was measured in medium and fibroblast cell lysates (Fig. 2). In patient 1, no neutralizing effects of anti-rhGAA antibodies had been observed at the ages of 0.4 and 3.2 years (activity in cell lysates 100 and 83% compared to the activity in controls [16]). At age 6.4 years enzyme activity in cell lysates was 49.7%. In patient 2, enzyme activity in cell lysate decreased from 79.8% at the age of 2.1 years to 60% at the age of 2.7 years [23]. In patient 3 enzyme activity in cell lysate was 93.7% at age of 2.1 years.

Figure 2 shows the effects of immunomodulation on neutralizing effects. At the ages of 7.6 years (patient 1), 3.6 years (patient 2), and 2.5 years (patient 3), no neutralizing effects of anti-rhGAA antibodies were observed respective enzyme activity in cell lysates of 145, 165, 147%).

### Clinical outcome measures

**Fig. 3:**
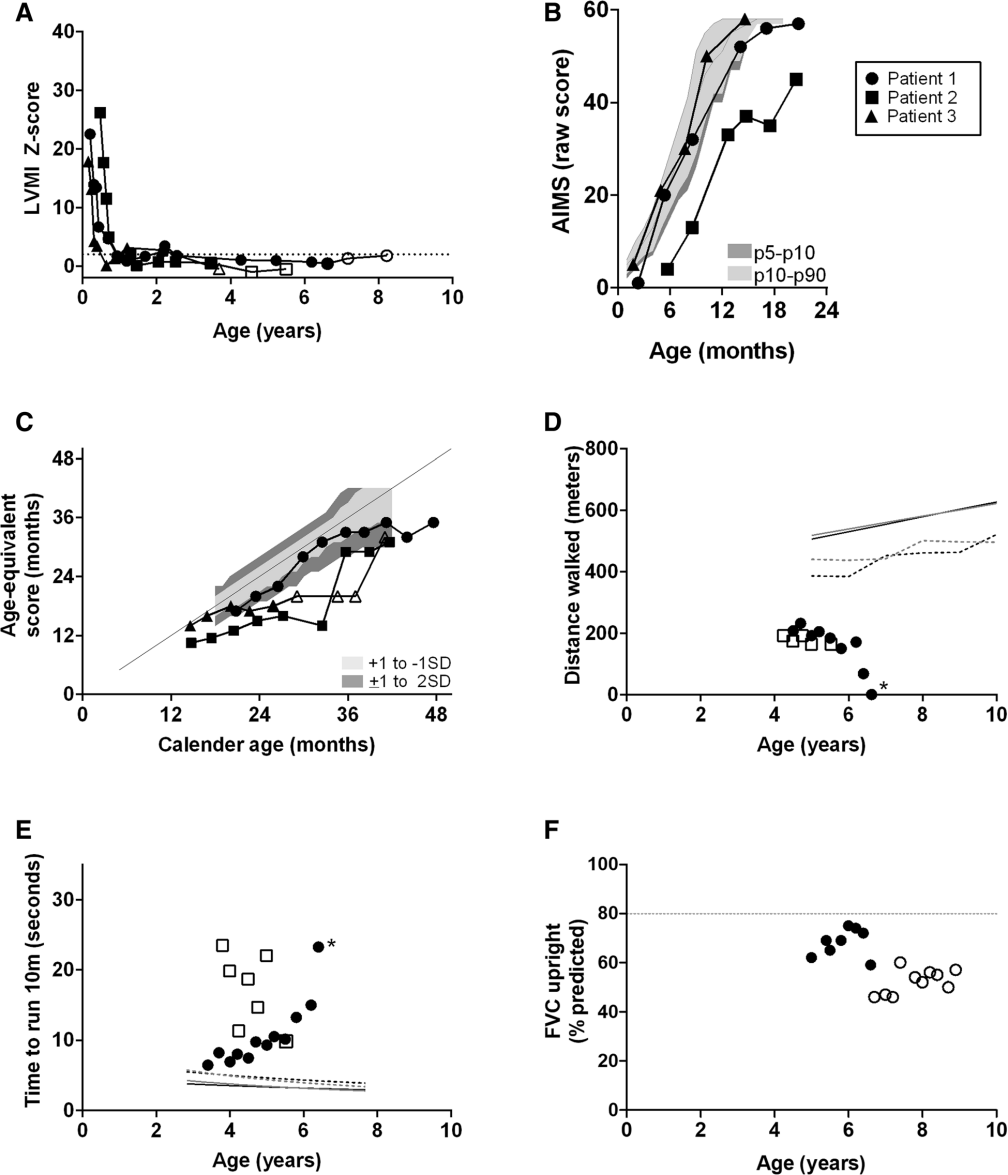
Clinical outcome. All closed symbols represent measurements taken before secondary immunomodulation; all open symbols represent measurements taken after immunomodulation, Circles: Patient 1, Squares: Patient 2, Triangels: Patient 3 **a**. AIMS score per patient during follow-up. Grey areas represent normal values. **b**. BSID II age-equivalent score during follow-up. **c**. Distance walked during 6-min walk test in patients 1 and 2, patient 3 is too young to perform the 6MWT. Patient 1 lost the ability to walk at the age of 6.6 years (marked with asterisk). **d**. Time to run 10 m in patients 1 and 2. Patient 1 lost the ability to perform this test at the age of 6.1 years (marked with asterisk). **e**. LVMI Z-score during follow-up. **f**. FVC % of predicted relate to age in patient 1. Patients 2 and 3 are too young to perform a pulmonary function test

At study end, all patients were alive, none required ventilatory support (Table 1), and LVMI was within normal limits (Fig. 3a). Patient 1 had lost the ability to walk before start of immunomoulation (Fig. 3d) and did not regain it. Patients 2 and 3 maintained the ability to walk. Figure 3b-e shows the motor performance of all three patients (AIMS, BSID II, 6MWT) and time they needed to run 10 m) before (closed symbols) and after start of secondary immunomodulation (open symbols). At the end of the study period, when patient 3 was aged 3.8 years, her BSID II scores were within normal limits; before, they had been slightly lower. It should be noted that around this age, all three patients—including those who had not yet started secondary immunomodulation—had had similar age-equivalant scores. Patient 3 was too young to perform the 6MWT. For patient, 2 the distance walked during the 6MWT remained stable over a period of 1.5 years. Pulmonary function tests could be performed only in patient 1 (Fig. 3f): before immunomodulation, the percentage of predicted of the forced vital capacity (FVC) had ranged between 59 and 75%; after immunomodulation it ranged from 46 and 60%.

### Safety and effects on IARs

Before start of secondary immunomodulation, all patients had experienced IARs (Table 1). After the start of secondary immunomodulation, no IARs were observed. Immunomodulation was well tolerated. None of the patients experienced serious infectious diseases. Mild erythema was observed at the Bortezomib injection site in all patients. No other adverse events were reported. At last observation, all patients were still receiving daily Rapamycin and monthly IVIG. Over the course of two to three months after the start of secondary immunomodulation, AST, ALT and CK levels all increased in the three patients, the increase in CK levels being the most prominent (from 842 to 3175 U/l (patient 1); from 1537 to 2939 U/l (patient 2); and from 3451 to 5363 U/l (patient 3), Additional file 1). The increase in CK levels remained unexplained.

## Discussion

In this study, we evaluated the effect of a secondary immunomodulation protocol using Rituximab, Bortezomib, Rapamycin and IVIG, to improve or stabilize clinical outcome of three classic infantile patients with high sustained antibody titers. We observed B-cell depletion and recovery in all three patients. Before immunomodulation anti-rhGAA antibody titers ranged from 1:156,250 to 1:781,250. At last assessment titers ranged from 1:31.250 to 1:156.250. Thus, secondary immunomodulation did not eliminate anti-rhGAA antibody titers. The neutralizing effects of anti-rhGAA antibodies that were observed in two patients before start of immunomodulation disappeared. Before secondary immunomodulation, all patients had experienced IARs; afterwards, none did so. It is noteworthy that none experienced serious infectious diseases during immunomodulation. We speculate that IVIG administrations may have contributed to the low infection rate.

It was difficult to fully judge the effect of immunomodulation on clinical outcome parameters. The two CRIM-negative patients who could walk at start maintained this ability. The patient who had lost this ability did not regain it. We observed some positive impact on the clinical stability.

### Overview of the literature on secondary immunomodulation: Effect on antibodies

To compare our results with those reported in the literature for the other patients with high titers receiving immunomodulation [19, 20, 25–28], we have summarized all reported data (8 patients) in Table 2. The first to publish their results were Messinger et al., who reported on two CRIM-negative patients receiving secondary immunomodulation with RTX, MTX and IVIG [19]. These patients’ anti-rhGAA antibody titers were substantially lower than those in our patients (maximum titer 1:12,800). The patients remained antibody-free after B-cell recovery.

**Table 2:** Overview of the literature on immunomodulation in classic infantile Pompe patients after antibodies have formed

Study	Pt	CRIM	Age at start of ERT	Age at start of IM	IM protocol	IM duration	Current ERT dose^a^	Follow-up since start of IM	Alive	Vent. free	Walks at study end	Titer at start of IM	Number of RTX infusions	B-cell recovery	Last known titer (time since B-cell recovery)
Messinger 2012 [19]	1	Neg	7 w	0.5 y	1	40 m	20 eow	4.6 y	Yes	No	No	1:1600	15	Yes	0 (20 m)
	2	Neg	16 d	2 m	1	IVIG ongoing	40 w	3 y	Yes	Yes	Yes	1:12,800	14	Yes	0 (10 m)
Banugaria 2012 [25]	1	Neg	4.2 m	8.8 m	2	RTX twice	variable	2.5 y	No	No	No	1:25,600	6	No	1:102,400 (before recovery)
Markic 2013 [27]	1	Pos	5 m	17.5 m	1	46 w	20 eow	3 y	Yes	No	No	1:6400	7	Yes	0 (unknown)
Deodato 2013 [26]	1	Neg	7 m	13 m	3	3 w	20 eow	22 m	Yes	No	No	1:3200^c^	1	Yes	1:100 (16 m)
Stenger 2015 [20]	1	Pos	23 d	11 m	4	Ongoing	20 eow	13 m	Yes	No	No	1:204,800	11	No	1:1200 (no recovery)
Kazi 2016 [28]	1	Pos	6.0 m	2.4 y	5A	3 y	40 w	5.5 y	Yes	No	No	1:204,800	19	Yes	0 (2.5 y)
	2	Neg	4.2 m	2 y	5B	Ongoing	40 eow	6.9 y	Yes	No	No	1:819,200	52	Yes	0 (4 w)
This study	1	Pos	2.4 m	6.6 y	6	Rap/IVIG ongoing	40 w	2.5 y	Yes	Yes	No^b^	1:156,250	3	No	1:31,250 (before recovery)
	2	Neg	5.8 m	3.5 y	6	Rap/IVIG ongoing	40 w	2.1 y	Yes	Yes	Yes	1:156,250^d^	3	Yes	1:31,250 (2 y)
	3	Neg	1.9 m	2.3 y	6	Rap/IVIG ongoing	40 w	1.5 y	Yes	Yes	Yes	1:781,250	3	Yes	1:156,250 (1.5 y)

^a^Excluding Banugaria 2012 (one patient) and the patients in our study, all patients started ERT dosed at 20 mg/kg every other week

^b^Patient did learn to walk, but lost the ability at the age of 6 years

^c^Titer was previously 1:25,400

^d^Titer was previously 1:800,000

*Pt* Patient, *CRIM* cross-reactive immunologic material, *Pos* Positive, *Neg* Negative, *ERT* enzyme replacement therapy, *w* weeks, *m* months, *y* years, *IM* immunomodulation, *eow* every other week, *RTX* Rituximab, *Vent. free* ventilator-free survival, *MTX* Methotrexate, *IVIG* intravenous immunoglobulin, *Rap* Rapamycin

Immunomodulation (IM) protocol used per study:

1. RTX 375 mg/m^2^/dose for 4 weekly iv doses followed by maintenance doses; MTX 0.5 mg/kg weekly oral doses; IVIG 500 mg/kg/month.

2. Cyclophosphamide 15 mg/kg iv on day 1 followed by 2 mg/kg/day iv for 9 days, IVIG 400 mg/kg day 5 through 9; Plasmapheresis day 1, 3 and 5 in week 20, 34 and 56. Between week 34 and 56 oral Cyclophosphamide 2 mg/kg was given. Followed by iv RTX 375 mg/m^2^/week in weeks 99 through 102 and in weeks 140 and 141.

3. Plasmapheresis on days 1, 3 and 5. RTX 375 mg/m^2^ iv once on day 7, directly followed by IVIG (dose not mentioned), with 4 extra IVIG doses over the following 8 months.

4. RTX 375 mg/m^2^/dose iv followed by 10 maintenance doses; Bortezomib 1 .3mg/m^2^/dose in 2 sessions of 4 iv doses. MTX 0.5 mg/kg for 27 oral doses; IVIG 500 mg/kg for 5 doses.

5. A Cyclophosphamide 250 mg/m^2^ iv twice; RTX 375 mg/m^2^/dose in 2 sessions of 4 doses followed by 11 maintenance doses; Bortezomib 1 .3mg/m^2^/dose in 3 sessions of 4 iv doses; MTX 15 mg/m^2^ oral doses; IVIG 400-500 mg/kg/month B RTX 375 mg/m^2^/dose for 4 iv doses followed by RTX maintenance doses 70 weeks later; Bortezomib 1 .3mg/m^2^/dose in 4 sessions of 4 iv doses; MTX 15 mg/m^2^ oral doses; IVIG 400-500 mg/kg/month.

6. RTX 375 mg/m^2^/dose for 3 iv doses; Bortezomib 1 .3mg/m^2^/dose for 6 iv doses. Rapamycin daily according to body weight from week 4 onwards; IVIG 500 mg/kg/month.

Subsequently Kazi et al. and Stenger et al. [20, 28] reported on the addition of Bortezomib to the Messinger protocol. They treated three patients with high anti-rhGAA antibody titers (1:200,000–1:819,200). Titers declined, or these patients were antibody-free at their last assessment (0–1:1200) [20, 28], a fourth patient, who received additional Cyclophosphamide instead of Bortezomib, continued to have high titers (increase from 1:25,600 to 1:204,800; 1:102,400 at last assessment) [25]. Markic et el. and Deodato et al. used slightly different protocols for two patients (one CRIM-negative and one CRIM-positive) with low anti-rhGAA antibody titers (1:6400 and 1:3200). After B-cell recovery titers remained low to undetectable [26, 27].

In our study, anti-rhGAA antibody titers in one CRIM-negative patient (patient 2) decreased substantially. Previously, he had received primary immunomodulation that had no effect on antibody titers [23]. When we decided that the same patient should start secondary immunomodulation at the age of 3.5 years, his titers were 1:800,000. An additional sample taken at the actual start of secondary immunomodulation was slightly lower (1:156,250) and declined further to 1:6250. It is unclear whether the decline in titers was due entirely to immunomodulation, or whether a decline was already in progress. At last assessment, the titer had increased to 1:31.250. In our two other patients, we observed limited effects on anti-rhGAA antibody titer. We conclude that the overall effect of secondary immunomodulation in our study was more limited than the effect of secondary immunomodulation in the other reports.

### Possible consequences of the different immunomodulation protocols used

It must be noted that different secondary immunomodulation protocols were used, and that it is not yet clear how the differences between them may explain the differences in anti-rhGAA antibody formation and elimination. Seven of the eight patients reported in the literature received an initial round of weekly RTX infusions [19, 20, 25, 27, 28]; thereafter, six of these patients continued to receive repeated RTX infusions every four to 12 weeks, to a maximum of 52 doses [19, 20, 27, 28]. RTX is a chimeric monoclonal antibody that induces apoptosis of CD20-expressing B cells, but does not eliminate memory B cells. To eliminate memory B cells, Bortezomib was added to the protocol, with Rapamycin to modulate T-cell responses and IVIG to overcome the period of immunoglobulin depletion. In addition, Rapamycin may have an impact on glycogen storage by influencing the mTOR pathway and inhibition of glycogen synthase [35]. It is possible that longer and/or more frequent dosing of RTX or Bortezomib could be more effective in preventing immune responses.

We also conclude that there are differences in the definition of and when to start immunomodulation. The definition may also be influenced by slight differences between the antibody assays used. In our earlier studies we did not find inhibitory effects in patients with titers below 1:31,250 [16]. Future research should seek to identify the most successful secondary immunomodulation protocol in patients with high sustained titers.

### Overview of the literature on secondary immunomodulation: Clinical outcome

As Table 2 shows, there are wide variations between the clinical outcome reported for patients receiving secondary immunomodulation. While seven of the eight patients reported in the literature (87.5%) were alive at study end, only one (12.5%) remained ventilator-free and learned to walk (patient 2 of Messinger et al.). This patient was the youngest at start of ERT (age 16 days), and at study end was receiving 40 mg/kg/week of ERT.

In our patients, the overall clinical outcome was better. Despite the development of high anti-rhGAA antibody titers, both of our CRIM-negative patients learned to walk and had survived ventilator free even before the start of secondary immunomodulation at the ages of 2.3 and 3.5 years. Banugaria et al. reported that it is very unlikely that CRIM negative patients with high sustained antibody titers survive ventilator free beyond the age of 2 years [21].

An important aspect of our study was that our patients received a higher ERT dose of 40 mg/kg/week from start of ERT. With a higher dosage, more antibody-free rhGAA should be available, and the neutralizing effects of the same titer are likely to be less severe than in patients receiving 20 mg/kg every other week.

According to earlier estimates, as much as 54% of the administered enzyme (about 10 mg/kg) is antibody-bound at a dose of 20 mg/kg and a titer of 1:156,250 [16]. Theoretically, if a similar amount (10 mg/kg) were bound upon administration of 40 mg/kg, about 30 mg/kg would still be available for uptake in the target tissues.

This may explain the overall better clinical outcome in our CRIM-negative patients. After start of secondary immunomodulation—which had been initiated due to a decline in the quality of movements—one patient improved on time tests, and the other, aged 3.8 years, performed within the normal limits of the BSID II, even though she had previously shown some deviation. The CRIM-positive patient, who had lost the ability to walk, stabilized.

In our study we were not able to eliminate antibodies. We believe, however, that high rhGAA antibody titers and, specifically the presence of neutralizing antibodies, are relevant to a patient’s outcome. We also believe that providing an adequate dose of rhGAA is just as important.

## Conclusion

While, to some extent, the immunomodulation protocol used in our study reduced antibody titers, it did not eliminate them. None of the patients experienced serious infections and occurrence of IARs disappeared. Increases in CK levels remained unexplained. Overall, there have been few reports on secondary immunomodulation, and various protocols have been applied. Future research should seek to identify the most successful secondary immunomodulation protocol in patients with high sustained titers.

## Additional file

Additional file 1: **Figure S1.** CK values measured over time for patient 1 (circle), patient 2 (square) and patient 3 (triangle). Closed symbols represent CK values taken before secondary immunomodulation; all open symbols represent measurements taken after immunomodulation. (TIF 778 kb)

## Abbreviations

6MWT: 6-min walk test
AIMS: Alberta Infant Motor Scale
ALT: Alanine Transaminase
AST: Aspartate Transaminase
BSID-II: Bayley Scales of Infant Development II
CK: Creatine Kinase
CRIM: Cross-reactive immunologic material
ELISA: Enzyme-linked immunosorbent assay
ERT: Enzyme replacement therapy
FVC: Forced vital capacity
GAA: Acid-α-glucosidase
HCM: Hypertrophic cardiomyopathy
IARs: Infusion-associated reactions
Ig: Gammaglobulin
IVIG: Intravenous Immunoglobulin
LVMI: Left-ventricular mass index
MTX: Methotrexate
NGT: Nasogastric tube
rhGAA: Recombinant human alpha-glucosidase
RTX: Rituximab
