# Supplementary figures and images for: Effects of immunomodulation in classic infantile Pompe patients with high antibody titers
Source: Orphanet J Rare Dis. 2019 Mar 22;14:71. doi: 10.1186/s13023-019-1039-z (PMC6431009; doi:10.1186/s13023-019-1039-z)

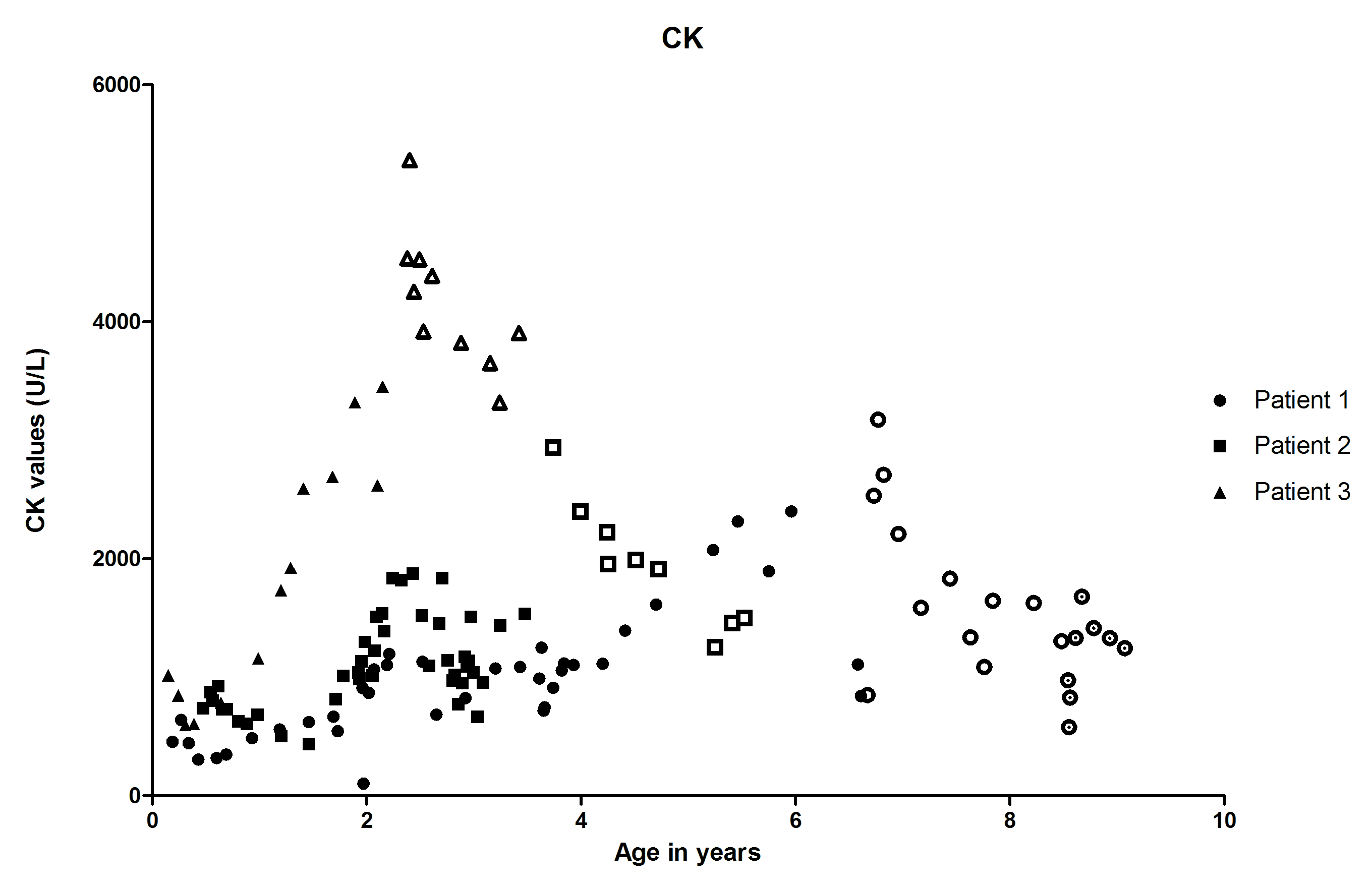

Supplement: Supplementary file 1 — Figure S1. CK values measured over time for patient 1 (circle), patient 2 (square) and patient 3 (triangle). Closed symbols represent CK values taken before secondary immunomodulation; all open symbols represent measurements taken after immunomodulation. (TIF 778 kb) [file 13023_2019_1039_MOESM1_ESM.tif]
